# The Effect of Periodontitis Severity on Diabetic Retinopathy: An Optical Coherence Tomography Study

**DOI:** 10.3390/diagnostics16050654

**Published:** 2026-02-24

**Authors:** Hatice Turkogullari, Gozde Nur Aydogan, Nur Yorgancilar, Oguz Kose, Huseyin Findik

**Affiliations:** 1 Department of Periodontology, Faculty of Dentistry, Recep Tayyip Erdoğan University, 53020 Rize, Turkey; 2 Department of Ophthalmology, Division of Surgical Medical Sciences, Faculty of Medicine, Recep Tayyip Erdoğan University, 53100 Rize, Turkey

**Keywords:** periodontitis, diabetic retinopathy, macular edema, optical coherence tomography, optical coherence tomography angiography

## Abstract

**Background**: The aim of this study was to comprehensively investigate the potential degenerative effects of periodontitis severity on retinal and choroidal structures in patients with different types of diabetic retinopathy (DR). **Materials and Methods**: The study’s Clinical Trials Registration Number is NCT07137013. A total of 100 participants (56 females and 44 males), each group consisting of 20 individuals, were allocated into five groups: systemically healthy controls (G1), diabetic patients without DR (G2: DM+ DR−), non-proliferative DR without diabetic macular edema (G3: NPDR DME−), non-proliferative DR with diabetic macular edema (G4: NPDR DME+), and proliferative DR (G5: PDR). Ocular examinations were performed using optical coherence tomography (OCT) and OCT angiography (OCTA). Retinal layer thicknesses, choroid-sclera interface (CSI), ganglion cell layer (GCL), retinal nerve fiber layer (RNFL), and peripapillary CSI were assessed by OCT, whereas superficial and deep retinal vessel densities and the foveal avascular zone (FAZ) were evaluated by OCTA. Clinical periodontal status was assessed using plaque index (PI), gingival index (GI), bleeding on probing (BOP), probing pocket depth (PPD), and clinical attachment loss (CAL). **Results**: In the G3 and G5 groups, the presence of stage III–IV periodontitis was associated with a marked increase in retinal layer thickness. GCL + Inner Plexiform Layer (GCL+) thickness was significantly reduced in individuals with stage III–IV periodontitis in almost all regions of the G5 group, except for the 3 mm nasal and inferior areas. Peripapillary CSI values showed a significant decrease with increasing periodontitis severity. RNFL thickness was significantly reduced in individuals with stage III–IV periodontitis, particularly in the G5 group. OCTA analyses demonstrated significant reductions in superficial and deep retinal vessel densities in several regions in the presence of stage III–IV periodontitis. Moreover, FAZ areas were significantly enlarged in individuals with stage III–IV periodontitis in the G2 and G5 groups. **Conclusions**: Periodontal inflammation, particularly in advanced periodontitis (stage III–IV), induces degenerative changes in the retinal microvasculature and neural tissues. Increasing periodontitis severity may represent a potential provoking factor in the pathogenesis of DR.

## 1. Introduction

Diabetic retinopathy (DR) is a common microvascular complication of diabetes mellitus (DM) and a major global public health concern, representing one of the leading causes of visual impairment among adults worldwide [1,2]. In DR, thickening of the retinal capillary basement membrane and increased vascular permeability occur, followed by progressive neovascularization and vascular proliferation within the retinal layers [3]. Contemporary imaging modalities are widely used to investigate structural and microvascular changes in the retina [4,5]. Among these, optical coherence tomography (OCT) and OCT angiography (OCTA) are currently considered gold-standard, non-invasive techniques for retinal assessment [6]. OCT enables high-resolution, contact-free measurement of retinal, choroidal, retinal nerve fiber layer (RNFL), and ganglion cell layer (GCL) thicknesses, whereas OCTA allows layer-by-layer visualization of retinal plexuses and quantitative evaluation of microvascular alterations at the capillary level [5,7]. Together, these modalities provide valuable insights into retinal structural and vascular changes in systemic diseases such as DM and periodontitis [8,9,10].

The pathogenesis of DR is primarily driven by chronic hyperglycemia, oxidative stress, inflammatory cytokine release, and endothelial dysfunction [11]. Periodontitis is likewise a chronic inflammatory disease characterized by similar pathophysiological mechanisms and affects nearly half of the global population, with a prevalence exceeding 60% among individuals aged ≥ 65 years [12,13,14,15]. DR is also more frequently observed in older individuals [16]. The association between DR and periodontitis can therefore be explained by shared pathways involving systemic inflammation, oxidative stress, and endothelial dysfunction [14]. Indeed, recent observational studies have suggested a potential link between these two diseases; however, investigations specifically addressing the impact of periodontal disease severity on retinal layer thickness in patients with DR remain limited [8,9,17,18,19,20].

In this context, the hypothesis that periodontal inflammation-induced systemic inflammatory burden may lead to microvascular alterations in the retina, thereby affecting retinal layer thickness, provides an important conceptual framework for explaining the association between DR and periodontitis. Therefore, the present study aimed to evaluate the relationship between periodontitis severity and retinal layer thickness in individuals with different types of DR using OCT and OCTA, and to comprehensively assess the potential effects of periodontal disease on retinal microstructures.

## 2. Materials and Methods

### 2.1. Ethical Approval

This cross-sectional study was conducted between February and June 2025 with the participation of 100 individuals aged ≥ 60 years (56 females and 44 males) who presented to the Department of Ophthalmology, Faculty of Medicine, Recep Tayyip Erdoğan University (RTEU). The study protocol was approved by the RTEU Non-Interventional Clinical Research Ethics Committee (Approval No: 2025/59) and was carried out in accordance with the principles of the Declaration of Helsinki. The study details were explained to all participants, and both written and verbal informed consent were obtained prior to enrollment. The study’s Clinical Trials Registration Number is NCT07137013.

### 2.2. Study Groups

Participants who met the inclusion criteria were allocated into five groups, each consisting of 20 individuals: Systemically healthy controls (G1), diabetic patients without DR (G2: DM+ DR−), patients with non-proliferative DR without diabetic macular edema (G3: NPDR DME−), patients with non-proliferative DR with diabetic macular edema (G4: NPDR DME+), and patients with proliferative DR (G5: PDR). All ophthalmologic diagnoses were established by an experienced ophthalmologist (H.F.).

According to the current periodontal disease classification [15], participants were further categorized into two groups based on periodontitis severity: stage I–II and stage III–IV.

A diagnosis of DM required an HbA1c level ≥ 6.5% [21]. Individuals with autoimmune diseases, osteoporosis, malignancy, those using immunosuppressive agents, oral contraceptives, or bisphosphonates, pregnant individuals, patients with vitreoretinal, optic nerve, or choroidal vascular diseases, cataract, glaucoma, retinal degeneration, uveitis, Behçet’s disease, or scleritis, a history of refractive or intraocular surgery, active infectious diseases (e.g., acute hepatitis, tuberculosis, AIDS), chronic medication use affecting periodontal tissues (e.g., cyclosporine A, phenytoin), or antioxidant supplementation were excluded from the study.

### 2.3. Sample Size Calculation

Sample size estimation was performed using G*Power (ver.3.1.9.7) software. Based on an effect size (d) of 0.40, a type I error rate (α) of 0.05, and a statistical power of 90% (1 − β), the total required sample size was calculated as 100 participants, with at least 20 individuals per group.

### 2.4. Examiner Calibration

Calibration for OCTA foveal avascular zone (FAZ) measurements and clinical periodontal measurements was performed by four examiners (H.F. and H.T., G.N.A, N.Y., respectively) in 10 individuals (5 with stage I–II and 5 with stage III–IV periodontitis) who were not included in the study, at two time points (baseline and 2 weeks later). The intraclass correlation coefficients were 0.969 for FAZ measurements and 0.978 for probing pocket depth (PPD), indicating excellent reproducibility. With a 99% confidence interval, these results were considered highly reliable (*p* < 0.0001).

### 2.5. Clinical Periodontal Measurements

Plaque index (PI) [22], gingival index (GI) [22], bleeding on probing (BOP) [23], PPD [24], and clinical attachment loss (CAL) [24] were recorded using a Williams periodontal probe (Hu-Friedy, Chicago, IL, USA). PPD was defined as the distance between the gingival margin and the deepest point of the periodontal pocket, and CAL as the distance between the cemento-enamel junction and the base of the pocket. Measurements were obtained at six sites per tooth (mesiobuccal, mid-buccal, distobuccal, mesiolingual, mid-lingual, and distolingual) for all teeth except third molars. PI and GI were assessed at four sites (mesial, distal, buccal, and palatal).

### 2.6. Optical Coherence Tomography Measurements

Prior to OCT imaging, pupil dilation was performed in all participants. Measurements were obtained by an experienced ophthalmologist (H.F.) using swept-source OCT and OCTA (DRI OCT Triton; Topcon, Tokyo, Japan).

**Figure 1:**
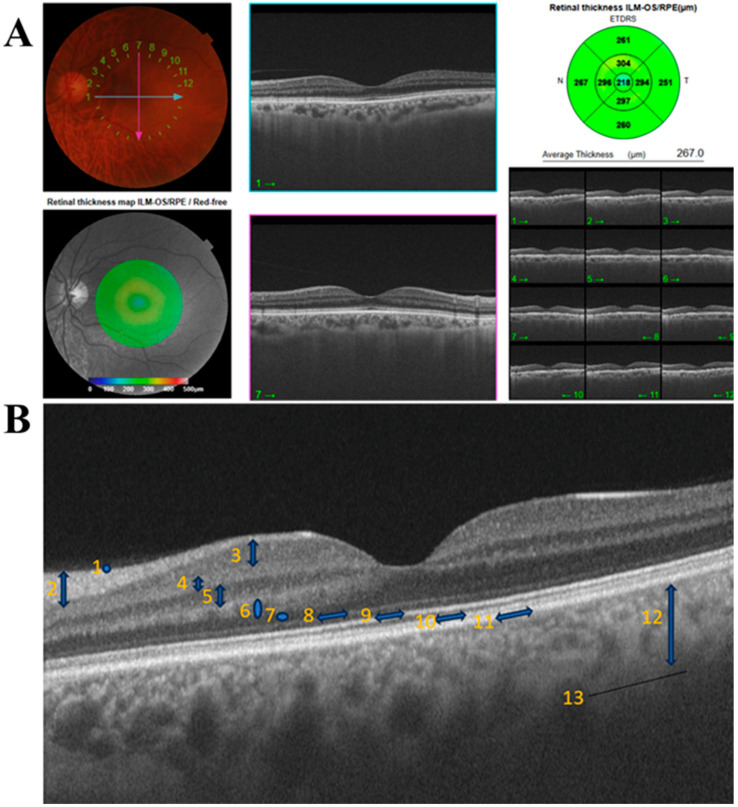
(**A**) Retinal Layer Thickness Measurement by OCT (**B**) Retina and Choroid Layers (1: Inner Limiting Membrane (ILM); 2: Retinal Nerve Fiber Layer (RNFL); 3: Ganglion Cell Layer (GCL); 4: Inner Plexiform Layer (IPL); 5: Inner Nuclear Layer (INL); 6: Outer Plexiform Layer (OPL); 7: Outer Nuclear Layer (ONL); 8: Outer Limiting Membrane (OLM); 9: Ellipsoid Zone; 10: Interdigitation Zone; 11: Rpe/Bruch’s Complex; 12: Choroid; 13: Choroid Sclera Interface (CSI)).

Macular thickness was evaluated using a nine-sector map centered on the fovea according to the Early Treatment Diabetic Retinopathy Study (ETDRS) (Bethesda, MD, USA) grid. The central 1 mm circle represented the foveal area, the surrounding 3 mm ring corresponded to the inner retinal layers, and the outer 6 mm ring represented the outer retinal layers (Figure 1A).

Based on the ETDRS grid, retinal layer thickness, choroid–sclera interface (CSI), GCL+ (GCL + inner plexiform layer (IPL)), and RNFL thicknesses were measured in nine regions. In addition, peripapillary CSI (BM–CSI) thickness was measured in four quadrants (superior, inferior, nasal, and temporal) using grid mode 4 [25] (Figure 1B). RNFL total thickness was defined as the global average thickness obtained from a circular peripapillary scan and automatically calculated by the OCT device as the mean value of the superior, inferior, nasal, and temporal quadrants. Measurements from the right and left eyes were averaged to obtain a single subject-level value for each parameter, and all statistical analyses were conducted using average data.

### 2.7. Optical Coherence Tomography Angiography Measurements

OCTA was used to assess vessel density and FAZ parameters in both superficial and deep retinal layers. A 6 × 6 mm scan area was applied for all images. The superficial retinal layer was defined as the region from 2.6 to 15.6 μm from the inner limiting membrane (ILM), and the deep retinal layer as the region from 15.6 to 70.2 μm from the ILM.

**Figure 2:**
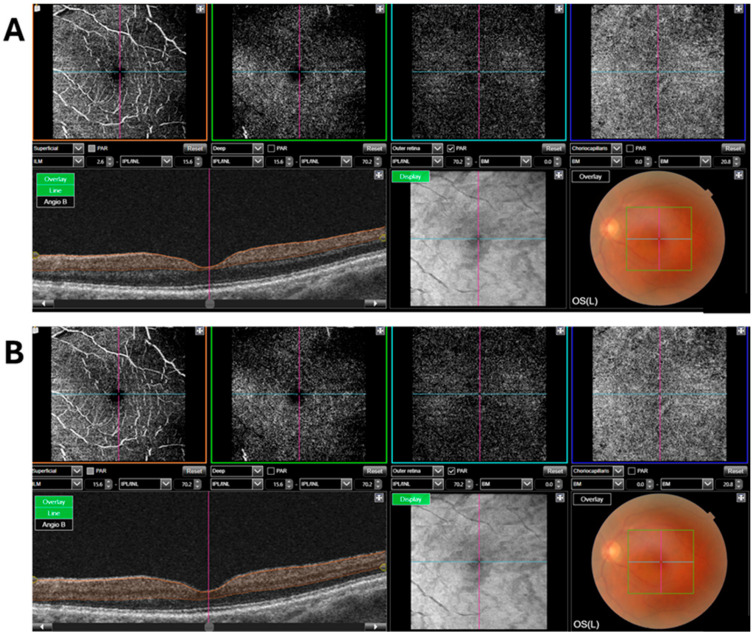
Optical Coherence Tomography Angiography (OCTA) Measurements of Retinal Vessel Density (**A**) Superficial Retinal Layer and (**B**) Deep Retinal Layer.

Vessel density was measured in the foveal area (central 1 mm diameter) and the parafoveal ring (1–3 mm), which was further divided into four quadrants: superior, inferior, nasal, and temporal (Figure 2).

The central retinal region in which no vascular structures were detectable was defined as the FAZ. The FAZ area was manually measured in all patients. Two reference lines passing through the center of the FAZ were drawn on each image: one along the superior-inferior axis (vertical) and the other along the temporal-nasal axis (horizontal). These lines symmetrically divided the FAZ into four quadrants, thereby establishing a central reference framework. Using these reference axes, the inner boundary of the FAZ was carefully delineated manually according to the points where the surrounding capillary network began. During this process, the innermost points of the capillary loops bordering the vessel-free zone were used as reference points for boundary delineation. The resulting closed contour was subsequently measured by the analysis software to calculate the FAZ area.

### 2.8. Statistical Analysis

Statistical analyses were performed using SPSS Statistics version 26 (IBM Corp., Armonk, NY, USA). The normality of the distribution of continuous variables was assessed using skewness and kurtosis values. Since parametric assumptions were satisfied, independent samples *t*-tests were used for comparisons between two groups, and one-way analysis of variance (ANOVA) was applied for comparisons among three or more groups. The chi-square (χ^2^) test was used to analyze categorical variables such as age and sex. *p* value < 0.05 was considered statistically significant.

## 3. Results

### 3.1. Demographic Findings

The study population consisted of 56 females and 44 males. 57 participants were in the 60–65 age range, and 43 were 66 years and older. Chi-square analysis revealed no statistically significant differences in gender (χ^2^ = 1.440; *p* = 0.230) or age (χ^2^ = 1.96; *p* = 0.162) distributions. When the distribution of participants according to the five study groups (G1–G5) was examined, it was observed that the ratio of women to men (χ^2^ = 3.409, *p* = 0.492) and the age distribution (χ^2^ = 1.877, *p* = 0.758) were relatively balanced in each group. This indicates that the groups were demographically homogeneous (Table 1) (Supplementary Table S1) (Changes in HbA1c values are presented in Supplementary Table S1).

### 3.2. Association Between Retinal Layer Thickness and Periodontal Disease Severity

With increasing periodontitis stage, significant increases were observed in the 1 mm central, 3 mm superior and temporal, and 6 mm inferior retinal layer thicknesses in the G4 and G5 groups. In contrast, in the G3 group, negative correlations with periodontitis stage were detected in the 1 mm central and 6 mm temporal retinal layers. A statistically significant increase in the 6 mm nasal retinal layer thickness was observed only in the G5 group (*p* = 0.025) (Table 2).

### 3.3. Association Between CSI Values and Periodontal Disease Severity

In the G3, G4, and G5 groups, significant decreases were observed in the 1 mm central, 3 mm superior and nasal, and 6 mm nasal CSI layers as periodontitis stage increased. In addition, statistically significant reductions were detected in the temporal regions (3 mm and 6 mm) in the G3 and G5 groups. A significant decrease was observed in the 3 mm inferior region in the G3 group and in the 6 mm superior region in the G4 group. As periodontitis severity increased, a significant decrease was noted in the 6 mm nasal region in the G2 group, whereas a significant increase was detected in the 6 mm inferior region in the G1 group (*p* = 0.012) (Table 3).

### 3.4. Association Between GCL+ Values and Periodontal Disease Severity

In the G5 group, statistically significant reductions were observed in almost all regions, except for the 3 mm nasal and inferior areas. Moreover, thinning the 1 mm central GCL+ was detected in the G3 and G4 groups with increasing periodontitis stage (Table 4).

### 3.5. Association Between RNFL Values and Periodontal Disease Severity

In the G5 group, statistically significant decreases were observed across all regions as periodontitis stage increased. Additionally, in the superior RNFL, a significant decrease was detected in the G4 group, whereas a significant increase was observed in the G1 group (Table 5).

### 3.6. Association Between Peripapillary CSI (BM–CSI) Values and Periodontal Disease Severity

In all groups, a statistically significant decrease in nasal CSI was detected with increasing periodontitis stage. In the superior and temporal CSI regions, significant reductions were observed in all groups except for G1. In the inferior CSI region, a statistically significant decrease associated with periodontitis severity was observed only in the G5 group (*p* = 0.049) (Table 6).

### 3.7. Association Between Superficial Retinal Layer Vessel Density (ILM 2.6- IPL/INL15.6) and Periodontal Disease Severity

As periodontitis stage increased, statistically significant decreases in superficial retinal layer vessel density were observed in the central region in the G3 group, the inferior region in the G4 group, and the nasal region in the G5 group (Table 7).

### 3.8. Association Between Deep Retinal Layer Vessel Density (ILM 15.6-IPL/INL70.2) and Periodontal Disease Severity

In the G3 and G4 groups, significant decreases in vessel density were recorded in the deep central and temporal regions with increasing periodontitis stage. In addition, a similar decrease was observed in the deep temporal region in the G2 group. The reduction in vessel density was statistically significant in the deep inferior region in the G4 group and in the deep superior region in the G5 group (Table 8).

### 3.9. Association Between FAZ (Superficial and Deep) Values and Periodontal Disease Severity

In both superficial and deep FAZ measurements, a statistically significant increase was detected in the G2 group as periodontitis stage increased. Furthermore, in the G5 group, a significant increase was observed only in the deep FAZ (Table 9).

## 4. Discussion

The association between periodontitis and DR is primarily attributed to shared mechanisms involving systemic inflammation, oxidative stress, and endothelial dysfunction [12,13,14,17,18,19]. In diabetes, hyperglycemia-induced overproduction of reactive oxygen species and the accumulation of advanced glycation end products (AGEs) promote endothelial dysfunction, which underlies the development of microvascular complications. This process is closely linked to the vascular damage and heightened inflammatory response observed in DR [3,26]. Similarly, periodontitis, as a chronic source of oral inflammation, increases the systemic inflammatory burden through the release of pro-inflammatory mediators such as Tumor necrosis factor (TNF)-α, Interleukin (IL)-6, and C-reactive protein into the circulation, thereby potentially impairing endothelial function [12,27]. These mechanisms may aggravate diabetic microangiopathy and facilitate the progression of DR. Moreover, periodontitis-related systemic inflammation may contribute to pathological retinal neovascularization by enhancing Vascular Endothelial Growth Factor expression secondary to retinal hypoxia [12,13,14]. In this cross-sectional study, we comprehensively evaluated the relationship between periodontal disease severity and retinal structural changes using advanced imaging modalities. OCT and OCTA enabled the assessment of retinal thickness, CSI, GCL, RNFL, peripapillary CSI, as well as superficial and deep retinal vessel densities and FAZ areas. To our knowledge, this is the first study to demonstrate, in a layer-specific manner, the potential impact of periodontal disease severity on both retinal microvasculature and neural tissues. The findings offer a novel interdisciplinary biomarker perspective for understanding how oral inflammation is reflected in systemic microvascular structures.

In the present study revealed significant structural alterations in retinal, choroidal, and neural layers with increasing periodontal disease severity in patients with DR. The increase in retinal thickness observed particularly in the G4 and G5 groups among individuals with stage III–IV periodontitis, together with regional thinning in the G3 group, indicates that periodontal inflammation extends beyond a localized oral pathology and may influence retinal microvascular structures via systemic pathways. Consistent with our findings, a previous study evaluating intraretinal layer thickness reported significant intergroup differences across all macular regions, with thinning in DM without DR and mild DR, and thickening in moderate DR, likely reflecting edema-related changes (*p* < 0.001) [28]. Similarly, we observed retinal thinning in the G2 group compared with G1, whereas thickening associated with edema and proliferative changes was evident in advanced DR stages.

In DR, pathological changes including disruption of retinal vascular structures, increased blood-retina barrier permeability, and ischemia-induced microaneurysms, edema, exudation, and neovascularization extend beyond the retina and also affect choroidal thickness and volume [29,30,31]. The choroid, located beneath the retinal pigment epithelium and characterized by rich vascularization, plays a critical role in supplying the outer retinal layers [32]. Several studies have demonstrated a close relationship between DR severity and choroidal alterations [33,34,35,36]. In the cross-sectional study, choroidal thickness generally decreased with increasing periodontal disease severity, with only a few regions remaining unaffected in certain groups. This pattern may reflect a periodontitis-related systemic subclinical pro-inflammatory state. Fernández-Espinosa et al. [36] demonstrated an inverse relationship between increased retinal thickness and choroidal thickness within the underlying pathophysiological processes. Wang et al. [33] reported that in patients with type 2 diabetes and DR, choroidal thickness increased in certain regions, whereas a general thinning was observed as disease severity progressed. Supporting our findings, Lains et al. [34], found that choroidal thickness was significantly lower in patients with PDR compared with the control group. Consistent with previous studies, our findings further demonstrate, for the first time, that both retinal and choroidal layer thicknesses increase in the early stages of periodontitis and decrease in the advanced stages.

In our study, GCL and RNFL thickness progressively decreased with increasing periodontitis severity. The more pronounced alterations observed in the G5 group suggest that periodontal inflammation may interact with DR-related vascular dysfunction, thereby accelerating neurodegenerative processes. Consistent with our findings, Sung et al. [37] reported a progressive reduction in GC-IPL thickness across all retinal layers with advancing stages of DR, while another case–control study [38] demonstrated significantly reduced GC-IPL thickness in both patients with DM without DR and those with DR compared with healthy controls. [38] In a study that included control subjects, patients with DM without DR, and those with NPDR, and evaluated RNFL thickness [39], it was reported that thickness was significantly lower than in the control group only in the superior temporal region in the DM group and in the inferior temporal region in the NPDR group. Although our study is methodologically different, it is valuable in that it compares RNFL thickness according to the severity stages of periodontitis and also includes a proliferative group. Moreover, our findings indicate generally statistically significant reductions in RNFL thickness in the G5 group. In individuals with diabetes, pre-existing microangiopathy may amplify the effects of inflammatory mediators and thereby accelerate neurodegenerative processes [40]. These findings are consistent with the existing literature, suggesting that periodontitis may exert adverse effects on the retina through systemic inflammation and vascular dysfunction [41,42,43].

Interestingly, RNFL thickening was observed in individuals with stage III–IV periodontitis in the G1 group. This finding may reflect transient edema-like changes or increased local vascular permeability induced by periodontal inflammation, as suggested in previous studies [41,44,45]. Thus, in nondiabetic individuals, periodontitis may lead to edema-like reversible retinal changes rather than true neurodegeneration. Supporting this interpretation, Arslan et al. [8] reported increased RNFL thickness in individuals with advanced periodontitis compared with those with early stages. In line with the findings of Arslan et al., it is suggested that periodontitis may induce subclinical inflammatory responses, thereby affect the ocular microvasculature and potentially lead to measurable changes in RNFL thickness. Additionally, in this study, regions showing a significant decrease in peripapillary choroidal thickness also exhibited concomitant and similarly directed significant reductions in RNFL thickness. This finding indicates a positive and significant association between peripapillary choroidal thickness and RNFL thickness.

OCTA findings further demonstrated that increasing periodontal disease severity was associated with significant reductions in superficial and deep retinal vessel densities in specific regions. These results align with previous reports of decreased foveal vessel density in metabolic syndrome and periodontitis [8], reduced superficial capillary density in mild NPDR [46], and diminished deep plexus density in eyes with DME+ [47]. Studies comparing type 1 and type 2 diabetes have also shown significant alterations in both capillary plexuses [48], while others have documented superficial capillary loss in diabetic individuals [49]. Moreover, reductions in peripapillary vessel density with increasing DR severity [50] and associations between vascular density loss and disease duration and severity [48,51,52] further support our observations. The observation of significant increases in superficial and deep FAZ values, particularly in the G2 group [53,54], as the severity of periodontitis increases, indicates that the stage of periodontitis is a factor affecting the FAZ in the early stages of diabetes [8,9,55]. In advanced stages, damage to the retina over time, such as changes in retinal thickness, becomes more pronounced, potentially masking the subtle FAZ changes observed earlier.

This cross-sectional study has several important limitations. Firstly, the failure to evaluate right and left eye data separately [56] precluded the identification of potential interocular differences in retinal layer thickness. This methodological limitation may have led to the neglect of intraocular asymmetries, in addition to interindividual variability, thereby restricting both clinical interpretability and the scientific validity of the findings. Secondly, periodontal disease involves a complex pathophysiological process driven by both chronic inflammatory responses and microbial infection. Considering that these two key mechanisms may affect retinal tissues through distinct biological pathways, evaluating their effects without distinguishing between the inflammatory and infectious components may complicate the biological interpretation of our data. Thirdly, the relatively small sample size and limited number of subgroups restrict the generalizability of the results. Furthermore, the relatively high standard deviations observed in some variables and the limited sample size in the subgroups may have reduced the statistical power to detect significant differences. Another significant limitation is that, because the study design was planned as an image-based cross-sectional analysis, systemic or local inflammatory biomarkers were not evaluated. Additionally, the lack of detailed treatment-related variables, concomitant systemic conditions, and inflammatory laboratory markers may have limited a more comprehensive assessment of potential confounding factors. Finally, the short follow-up period of the study did not allow comprehensive monitoring of structural changes that may occur over time in retinal and periodontal tissues and limited the evaluation of long-term effects. Therefore, future large-scale, long-term, and multicenter studies with larger sample sizes, incorporating multidisciplinary approaches that include systemic and local biomarkers as well as infectious mechanisms, are recommended to advance understanding in this critical field.

## 5. Conclusions

This cross-sectional study suggests that periodontal inflammation is associated with systemic microvascular dysfunction and may contribute to the pathophysiology of DR. The effects of periodontitis severity on retinal layer thickness and vascularization appear to be more pronounced in the advanced stages of DR.

## Figures and Tables

**Table 1:** Demographic Findings.

Variable	Category	Total N	G1 N (%)	G2 N (%)	G3 N (%)	G4 N (%)	G5 N (%)
**Gender**	Female	56	13 (65)	12 (60)	9 (45)	13 (65)	9 (45)
	Male	44	7 (35)	8 (40)	11 (55)	7 (35)	11 (55)
**χ^2^ (*p*)**		1.440 (0.230)	3.409 (0.492)				
**Age**	60–65 years	57	9 (45)	13 (65)	12 (60)	12 (60)	11 (55)
	≥66 years	43	11 (55)	7 (35)	8 (40)	8 (40)	9 (45)
**χ^2^ (*p*)**		1.960 (0.162)	1.877 (0.758)				
**Age (Mean ± S.D.)**		64.07 ± 6.90					

(G1: systemically healthy group; G2: patients with diabetes mellitus without diabetic retinopathy (DR); G3: patients with non-proliferative DR without diabetic macular edema; G4: patients with non-proliferative DR with diabetic macular edema; G5: patients with proliferative DR; N: frequency, %: percentage, SD: standard deviation, X^2^: one-sample chi-square test, *p* * < 0.05).

**Table 2:** Changes in Retinal Thickness.

	Group	Stage I–II		Stage III–IV		t	*p*
		Mean (µm)	S.D.	Mean (µm)	S.D.		
OU 1 mm retinal layer thickness	G1	233.08	18.99	234.36	17.7	−0.147	0.885
	G2	241.5	24.84	235.07	33.24	0.423	0.677
	**G3**	**281.94**	**58.4**	**253.08**	**34.89**	**3.125**	0.047 *
	**G4**	**283.54**	**54.64**	**333.63**	**70.59**	**−1.071**	0.029 *
	**G5**	**290.63**	**87.19**	**349.79**	**68.92**	**−0.539**	0.026 *
OU 3 mm superior retinal layer thickness	G1	299.58	25.32	311.36	19.72	−1.065	0.301
	G2	294.42	23.12	292.89	34.91	0.097	0.924
	G3	313	32.62	295.87	33.03	1.141	0.269
	**G4**	**304.06**	**13.07**	**331.96**	**16.14**	**−3.166**	0.026 *
	**G5**	**313.63**	**13.68**	**342.96**	**12.43**	**−2.908**	0.016 *
OU 3 mm inferior retinal layer thickness	G1	298.38	23.26	307.64	24.25	−0.837	0.414
	G2	270	39.13	282.04	38.99	−0.632	0.535
	G3	306.88	46.96	304.33	21.11	0.144	0.889
	G4	325.44	41.75	329.63	67.15	−0.157	0.877
	G5	319.63	75.08	322.33	47.8	−0.11	0.914
OU 3 mm nasal retinal layer thickness	G1	303.77	18.29	314.71	18.82	−1.264	0.222
	G2	293.92	22.63	293.61	31.89	0.021	0.983
	G3	304.37	47.73	301.33	16.39	0.174	0.866
	G4	312	41.82	322.29	30.61	−0.601	0.555
	G5	313.13	51.03	329.96	64.45	−0.558	0.584
OU 3 mm temporal retinal layer thickness	G1	288.62	20.18	296.57	21.5	−0.823	0.422
	G2	274.92	30.31	267.86	53.97	0.298	0.769
	G3	316.13	42.24	295.42	25.73	1.369	0.188
	**G4**	**305.06**	**16.3**	**332.88**	**10.28**	**−4.966**	0.034 *
	**G5**	**312.25**	**79.43**	**327.38**	**69.87**	**−0.442**	0.016 *
OU 6 mm superior retinal layer thickness	G1	266.85	25.51	275.14	15.53	−0.78	0.445
	G2	252.75	19.28	254.82	30.43	−0.153	0.88
	G3	283.56	20.21	273.13	22.8	1.047	0.309
	G4	275.94	40.97	298.29	50.01	−0.43	0.674
	G5	304.56	31.64	317.38	47.41	−0.442	0.664
OU 6 mm inferior retinal layer thickness	G1	260.88	18.7	264.36	13.2	−0.434	0.669
	G2	244.58	42.66	248.75	28.6	−0.412	0.685
	G3	271.44	47.11	269.96	23.38	0.094	0.926
	**G4**	**279.5**	**4.72**	**297.88**	**9.2**	**−3.109**	0.028 *
	**G5**	**288.75**	**8.95**	**321.88**	**13.56**	**−4.45**	0.012 *
OU 6 mm nasal retinal layer thickness	G1	280.62	19.29	288.14	12.3	−0.929	0.365
	G2	274.17	19.15	276.36	24.8	−0.258	0.799
	G3	284.38	34.25	284	16.93	0.033	0.974
	G4	290.94	31.92	293.38	18.12	−0.941	0.359
	**G5**	**292.44**	**11.99**	**313.96**	**9.56**	**−3.177**	0.025 *
OU 6 mm temporal retinal layer thickness	G1	255.65	17.2	257.07	16.23	−0.179	0.86
	G2	236.67	22.37	230.5	44.33	0.192	0.85
	**G3**	**286.38**	**23.15**	**262.67**	**20.3**	**2.356**	0.034 *
	G4	282.31	48.74	291.88	53.01	−0.573	0.574
	G5	296.5	74.17	316.96	51.53	−0.648	0.53

(OU: Oculus Uterque; mm: millimeters; µm: micrometers; G1: systemically healthy group; G2: patients with diabetes mellitus without diabetic retinopathy (DR); G3: patients with non-proliferative DR without diabetic macular edema; G4: patients with non-proliferative DR with diabetic macular edema; G5: patients with proliferative DR; OU values represent the mean of right and left eye measurements for each participant; SD: standard deviation; *p* * < 0.05).

**Table 3:** Changes in CSI Values.

	Group	Stage I–II		Stage III–IV		t	*p*
		Mean (µm)	S.D.	Mean (µm)	S.D.		
OU 1 mm CSI	G1	234.08	59.41	280.07	47.99	−1.756	0.096
	G2	213.42	81.11	219.5	61.61	−0.184	0.856
	**G3**	**284.94**	**58.17**	**212.71**	**55.42**	**2.772**	0.015 *
	**G4**	**262.5**	**19.78**	**223.67**	**28.38**	**1.99**	0.038 *
	**G5**	**264.38**	**18.57**	**216**	**29.6**	**2.13**	0.028 *
OU 3 mm superior CSI	G1	230.77	52.58	262.57	45.56	−1.347	0.195
	G2	221.42	72.47	224.07	56.08	−0.089	0.93
	**G3**	**262.13**	**15.01**	**214.38**	**20.47**	**1.927**	0.046 *
	**G4**	**260.88**	**15.34**	**206.29**	**32.64**	**1.903**	0.048 *
	**G5**	**247.06**	**31.14**	**220**	**17.5**	**3.23**	0.027 *
OU 3 mm inferior CSI	G1	219.04	54.17	260.86	46.84	−1.721	0.102
	G2	216.58	62.47	217.5	68.77	−0.028	0.978
	**G3**	**273.63**	**52.79**	**218.38**	**58.66**	**2.192**	0.043 *
	G4	251.81	65.26	206.92	62.22	1.535	0.146
	G5	257.88	65.41	225	84.76	0.926	0.367
OU 3 mm nasal CSI	G1	239.08	54.09	264.57	32.51	−1.133	0.272
	G2	213.67	73.79	207.61	75.11	0.167	0.871
	**G3**	**288.94**	**75.81**	**203.58**	**51.94**	**2.779**	0.017 *
	**G4**	**261.75**	**66.84**	**217.71**	**88.08**	**1.908**	0.043 *
	**G5**	**253.13**	**30.3**	**202.96**	**25.98**	**2.25**	0.034 *
OU 3 mm temporal CSI	G1	232.31	58.4	251.71	56.69	−0.716	0.483
	G2	209.25	79.51	216.61	72.96	−0.194	0.85
	**G3**	**257.19**	**49.46**	**207.25**	**50.89**	**2.093**	0.049 *
	G4	235.38	63.54	209.38	104.25	1.318	0.208
	**G5**	**252.94**	**81.65**	**209.17**	**52.67**	**2.19**	0.035 *
OU 6 mm superior CSI	G1	219.92	56.59	235.29	45.85	−0.615	0.546
	G2	211.58	45.12	208.11	55.42	0.135	0.894
	G3	240.13	43.22	192.67	58	1.691	0.115
	**G4**	**231.63**	**29.37**	**190.17**	**19.65**	**2.341**	0.019 *
	G5	224.19	70.36	204.21	85.73	0.569	0.577
OU 6 mm inferior CSI	**G1**	**196.23**	**48.58**	**253.86**	**32.82**	**−2.796**	0.012 *
	G2	198.42	72.93	190.82	74.21	0.211	0.835
	**G3**	**233.75**	**55.13**	**194.46**	**43.78**	**2.65**	0.016 *
	**G4**	**231.56**	**73.17**	**188.42**	**69.42**	**3.17**	0.012 *
	G5	224	29.55	206.92	41.43	0.474	0.642
OU 6 mm nasal CSI	G1	201.73	58.31	209.86	35.81	−0.334	0.742
	**G2**	**186.17**	**10.45**	**165.46**	**8.12**	**2.564**	0.041 *
	**G3**	**234.44**	**70.86**	**164.58**	**47.54**	**2.524**	0.021 *
	**G4**	**218.56**	**35.23**	**180.54**	**23.74**	**2.39**	0.038*
	**G5**	**219.75**	**89.52**	**168.13**	**66.61**	**3.28**	0.011 *
OU 6 mm temporal CSI	G1	192.27	52.25	222.64	61.66	−1.166	0.259
	G2	192.67	47.89	200.11	57.54	−0.277	0.785
	**G3**	**240.81**	**58.34**	**183.04**	**44.15**	**2.445**	0.032 *
	G4	213.13	64.84	191.13	108.26	0.568	0.577
	**G5**	**234**	**86.42**	**196.63**	**70.98**	**1.98**	0.045 *

(OU: Oculus Uterque; CSI: Choroid-Sclera Interface; mm: millimeters; µm: micrometers; G1: systemically healthy group; G2: patients with diabetes mellitus without diabetic retinopathy (DR); G3: patients with non-proliferative DR without diabetic macular edema; G4: patients with non-proliferative DR with diabetic macular edema; G5: patients with proliferative DR; OU values represent the mean of right and left eye measurements for each participant; SD: standard deviation; *p* * < 0.05).

**Table 4:** Changes in GCL+ Values.

	Group	Stage I–II		Stage III–IV		t	*p*
		Mean (µm)	S.D.	Mean (µm)	S.D.		
OU 1 mm GCL+	G1	44.54	8.37	44.71	10.42	−0.041	0.968
	G2	54.33	16.17	47.96	14.14	0.886	0.387
	**G3**	**67.25**	**17.11**	**48.42**	**15.31**	**2.292**	0.042 *
	**G4**	**70.25**	**6.25**	**61.71**	**8.79**	**2.45**	0.039 *
	**G5**	**67.75**	**12.4**	**57.92**	**13.29**	**1.98**	0.049 *
OU 3 mm superior GCL+	G1	86.31	10.13	91.57	9.52	−1.131	0.273
	G2	86.08	9.18	84.86	15.21	0.182	0.858
	G3	84.5	14.33	83.79	11.93	0.116	0.91
	G4	85.38	8.45	77.08	25.14	0.893	0.384
	**G5**	**86.75**	**12.43**	**76.17**	**8.93**	**2.19**	0.045 *
OU 3 mm inferior GCL+	G1	86.65	10.29	89.79	10.68	−0.641	0.53
	G2	81.5	11.1	79.79	16.64	0.27	0.791
	G3	85.25	18.03	82.88	10.93	0.368	0.717
	G4	80.19	19.28	81.88	16.82	−0.207	0.838
	G5	71.94	9.37	76.63	21.37	−0.67	0.513
OU 3 mm nasal GCL+	G1	87.92	9.44	93.86	8.66	−1.378	0.185
	G2	84.58	12.13	84.29	13.18	0.049	0.962
	G3	87.13	21.51	82.37	13.81	0.604	0.553
	G4	81.88	17.72	83	20.72	−0.13	0.898
	G5	79.56	7.49	78.21	21.36	0.202	0.843
OU 3 mm temporal GCL+	G1	81.65	10.18	86.36	9.85	−0.996	0.332
	G2	79.5	14.11	78.93	14.66	0.082	0.936
	G3	87.19	19.45	80.33	11	0.905	0.387
	G4	85.5	10.69	80.04	21.16	0.76	0.458
	**G5**	**85.75**	**14.52**	**74.33**	**10.25**	**2.15**	0.039 *
OU 6 mm superior GCL+	G1	62.46	6.87	63.71	5.38	−0.417	0.682
	G2	57.42	5.86	59.68	11.27	−0.461	0.651
	G3	63.56	8.19	64.38	10.13	−0.197	0.846
	G4	68.44	10.46	59.17	15.5	1.597	0.128
	**G5**	**68.56**	**15.02**	**57.67**	**10.54**	**2.29**	0.037 *
OU 6 mm inferior GCL+	G1	60.65	6.55	61.79	5.15	−0.395	0.698
	G2	59.58	17.41	59.07	10.8	0.588	0.564
	G3	67.75	11.87	61.08	10.69	1.28	0.221
	G4	64.19	21.05	62.75	12.35	0.174	0.865
	**G5**	**76**	**16.29**	**62**	**15.73**	**2.67**	0.035 *
OU 6 mm nasal GCL+	G1	66.12	6.96	67.57	5.49	−0.477	0.639
	G2	64.75	8.13	67.96	10.93	−0.081	0.936
	G3	64.81	12.14	67.71	9.44	−1.28	0.221
	G4	67.19	7.8	69.25	9.67	−0.526	0.606
	**G5**	**71.94**	**10.8**	**62.71**	**13.62**	**2.97**	0.028 *
OU 6 mm temporal GCL+	G1	67.96	6.67	68.93	7.08	−0.303	0.765
	G2	61.75	9.02	60.71	15.4	0.644	0.528
	G3	72.94	11.49	68.08	11.14	0.32	0.753
	G4	72.63	15.21	67.75	11.96	0.802	0.433
	**G5**	**80.56**	**12.01**	**63.46**	**14.49**	**2.869**	0.011 *

(OU: Oculus Uterque; GCL+: Ganglion Cell Layer plus Inner Plexiform Layer; mm: millimeters; µm: micrometers; G1: systemically healthy group; G2: patients with diabetes mellitus without diabetic retinopathy (DR); G3: patients with non-proliferative DR without diabetic macular edema; G4: patients with non-proliferative DR with diabetic macular edema; G5: patients with proliferative DR; OU values represent the mean of right and left eye measurements for each participant; SD: standard deviation; *p* * < 0.05).

**Table 5:** Changes in RNFL Values.

	Group	Stage I–II		Stage III–IV		t	*p*
		Mean (µm)	S.D.	Mean (µm)	S.D.		
OU TT RNFL	G1	99.65	12.81	109.86	5.55	−1.989	0.062
	G2	100.25	18.88	99.25	12.15	0.143	0.888
	G3	94.31	19.18	98.33	18.23	−0.474	0.642
	G4	98.31	16.94	94.71	15.56	0.49	0.63
	**G5**	**100.13**	**7.68**	**85.50**	**19.76**	**2.315**	0.035 *
OU superior RNFL	**G1**	**122.69**	**14.96**	**139.14**	**6.25**	**−2.755**	0.013 *
	G2	122.83	24.90	124.68	20.31	−0.174	0.863
	G3	119.79	21.56	107.75	28.27	1.082	0.294
	**G4**	**123.69**	**15.87**	**112.37**	**14.26**	**2.159**	0.042 *
	**G5**	**111.63**	**11.11**	**98.96**	**17.53**	**1.862**	0.041 *
OU inferior RNFL	G1	126.12	16.97	139.93	12.73	−1.879	0.077
	G2	128.67	25.90	125.29	16.79	0.351	0.73
	G3	118.19	30.32	123.50	26.50	−0.403	0.693
	G4	124.44	31.38	120.33	23.30	0.336	0.74
	**G5**	**122.37**	**11.68**	**106.92**	**17.07**	**2.16**	0.038 *
OU nasal RNFL	G1	80.69	16.32	87.43	8.07	−1.018	0.322
	G2	81.67	13.31	79.79	8.68	0.379	0.709
	G3	79.42	15.47	73.75	12.13	0.915	0.373
	G4	70.81	14.84	73.67	17.40	−0.38	0.708
	**G5**	**78.13**	**10.99**	**64.88**	**10.85**	**2.849**	0.022 *
OU temporal RNFL	G1	68.77	14.95	73.29	6.26	−0.757	0.459
	G2	71.75	10.59	67.29	11.25	0.826	0.419
	G3	73.88	13.11	70.88	26.08	0.342	0.736
	G4	74.19	16.46	72.42	13.70	0.262	0.797
	**G5**	**87.81**	**12.49**	**72.46**	**15.93**	**2.408**	0.027 *

(OU: Oculus Uterque; TT: Total Thickness; RNFL: Retinal Nerve Fiber Layer; µm: micrometers; G1: systemically healthy group; G2: patients with diabetes mellitus without diabetic retinopathy (DR); G3: patients with non-proliferative DR without diabetic macular edema; G4: patients with non-proliferative DR with diabetic macular edema; G5: patients with proliferative DR; OU values represent the mean of right and left eye measurements for each participant; SD: standard deviation; *p* * < 0.05).

**Table 6:** Changes in Peripapillary CSI (BM–CSI) Values.

	Group	Stage I–II		Stage III–IV		t	*p*
		Mean (µm)	S.D.	Mean (µm)	S.D.		
OU superior CSI	G1	148.27	69.68	175.43	37.50	−0.952	0.354
	**G2**	**168.33**	**21.94**	**130.61**	**32.08**	**2.373**	0.027 *
	**G3**	**167.31**	**31.81**	**137.83**	**33.92**	**2.40**	0.034 *
	**G4**	**168.74**	**80.15**	**130.41**	**69.72**	**2.30**	0.023 *
	**G5**	**185.64**	**65.09**	**147.09**	**57.09**	**2.40**	0.027 *
OU inferior CSI	G1	119.15	63.41	157.50	59.20	−1.319	0.204
	G2	127.75	50.83	110.11	65.67	0.584	0.566
	G3	112.94	42.51	132.63	74.45	−0.751	0.463
	G4	148.19	71.91	115.00	63.62	1.086	0.292
	**G5**	**163.75**	**38.12**	**132.17**	**31.67**	**1.97**	0.049 *
OU nasal CSI	**G1**	**121.73**	**45.21**	**187.14**	**53.52**	**−2.749**	**0** .019 *
	**G2**	**150.42**	**38.99**	**118.07**	**27.97**	**2.397**	0.023 *
	**G3**	**157.38**	**15.97**	**127.92**	**16.97**	**2.12**	0.032 *
	**G4**	**166.19**	**35.24**	**126.46**	**28.70**	**3.19**	0.017 *
	**G5**	**165.19**	**38.48**	**111.21**	**33.76**	**4.142**	0.012 *
OU temporal CSI	G1	150.15	54.77	152.50	67.31	−0.079	0.938
	**G2**	**161.75**	**30.65**	**129.82**	**23.77**	**1.90**	0.046 *
	**G3**	**178.38**	**25.98**	**143.29**	**20.57**	**2.98**	0.021 *
	**G4**	**183.69**	**22.04**	**128.46**	**34.57**	**4.23**	0.015 *
	**G5**	**185.43**	**38.45**	**145.27**	**35.48**	**2.30**	0.026 *

(OU: Oculus Uterque; CSI: Choroid–Sclera Interface; µm: micrometers; G1: systemically healthy group; G2: patients with diabetes mellitus without diabetic retinopathy (DR); G3: patients with non-proliferative DR without diabetic macular edema; G4: patients with non-proliferative DR with diabetic macular edema; G5: patients with proliferative DR; OU values represent the mean of right and left eye measurements for each participant; SD: standard deviation; *p* * < 0.05).

**Table 7:** Changes in Superficial Retinal Vessel Density.

	Group	Stage I–II		Stage III–IV		t	*p*
		Mean (%)	S.D.	Mean (%)	S.D.		
OU S central	G1	16.80	3.40	17.64	3.39	−0.527	0.605
	G2	18.42	2.52	19.15	4.48	−0.374	0.713
	**G3**	**20.49**	**5.34**	**16.40**	**3.40**	**2.107**	0.049 *
	G4	22.36	5.73	20.81	6.56	0.541	0.595
	G5	22.81	9.43	24.32	9.15	−0.357	0.725
OU S superior	G1	39.13	4.36	41.26	2.10	−1.208	0.243
	G2	37.56	5.35	38.39	4.28	−0.367	0.718
	G3	34.96	5.16	36.72	4.81	−0.776	0.448
	G4	36.06	2.33	36.42	5.28	−0.204	0.841
	G5	38.87	5.34	37.07	5.03	0.764	0.455
OU S inferior	G1	39.64	2.74	39.36	2.75	0.214	0.833
	G2	36.67	7.94	36.05	5.72	0.2	0.844
	G3	35.32	4.95	36.45	4.09	−0.536	0.601
	**G4**	**36.22**	**1.78**	**32.58**	**6.35**	**2.37**	0.045 *
	G5	34.02	4.05	35.21	4.48	−0.602	0.555
OU S nasal	G1	39.24	3.79	41.03	2.64	−1.103	0.285
	G2	37.56	4.84	37.40	4.02	0.078	0.939
	G3	35.72	6.11	36.09	4.34	−0.149	0.884
	G4	36.76	1.52	36.66	4.93	0.065	0.949
	**G5**	**36.93**	**2.38**	**34.06**	**1.13**	**2.465**	0.03 *
OU S temporal	G1	40.81	4.80	42.57	2.57	−0.891	0.385
	G2	40.92	4.44	38.68	4.61	1.007	0.327
	G3	38.37	2.10	37.91	3.85	0.305	0.764
	G4	37.96	1.60	36.20	6.69	0.874	0.398
	G5	38.74	2.42	37.25	3.76	1.079	0.295

(OU: Oculus Uterque; S: superficial; %: percentage; G1: systemically healthy group; G2: patients with diabetes mellitus without diabetic retinopathy (DR); G3: patients with non-proliferative DR without diabetic macular edema; G4: patients with non-proliferative DR with diabetic macular edema; G5: patients with proliferative DR; OU values represent the mean of right and left eye measurements for each participant; SD: standard deviation; *p* * < 0.05).

**Table 8:** Changes in Deep Retinal Vessel Density.

	Group	Stage I–II		Stage III–IV		t	*p*
		Mean (%)	S.D.	Mean (%)	S.D.		
OU D central	G1	15.46	3.22	16.51	3.44	−0.679	0.506
	G2	16.43	2.69	19.60	6.80	−1.093	0.289
	**G3**	**23.01**	**9.64**	**15.92**	**3.59**	**2.342**	0.031 *
	**G4**	**23.39**	**7.22**	**21.55**	**6.74**	**1.983**	0.047 *
	G5	25.72	10.51	25.28	9.66	0.097	0.924
OU D superior	G1	42.89	3.55	45.25	2.63	−1.54	0.141
	G2	40.35	6.49	39.86	5.88	0.166	0.87
	G3	37.80	5.12	38.81	5.96	−0.393	0.699
	G4	38.53	3.84	38.07	6.13	0.19	0.851
	**G5**	**40.35**	**1.62**	**38.80**	**1.51**	**1.99**	0.049 *
OU D inferior	G1	42.25	3.98	43.01	2.55	−0.454	0.655
	G2	40.67	7.51	38.12	6.51	0.768	0.453
	G3	38.05	5.91	38.10	4.94	−0.021	0.984
	**G4**	**37.57**	**3.47**	**34.28**	**5.95**	**2.11**	0.039 *
	G5	34.84	4.24	35.70	4.68	−0.428	0.674
OU D nasal	G1	42.95	5.17	45.71	2.63	−1.312	0.206
	G2	41.60	6.63	39.73	5.66	0.644	0.528
	G3	39.01	5.69	37.91	6.01	0.41	0.687
	G4	38.47	3.07	38.77	4.86	−0.151	0.882
	G5	36.82	3.26	35.39	5.87	0.699	0.493
OU D temporal	G1	42.98	6.44	45.48	2.80	−0.969	0.346
	**G2**	**42.32**	**2.55**	**40.51**	**2.10**	**1.97**	0.041 *
	**G3**	**41.27**	**1.55**	**39.87**	**1.27**	**2.14**	0.039 *
	**G4**	**39.24**	**1.85**	**37.21**	**1.67**	**2.328**	0.047 *
	G5	39.02	4.58	39.21	3.15	−0.103	0.919

(OU: Oculus Uterque; D: deep; %: percentage; G1: systemically healthy group; G2: patients with diabetes mellitus without diabetic retinopathy (DR); G3: patients with non-proliferative DR without diabetic macular edema; G4: patients with non-proliferative DR with diabetic macular edema; G5: patients with proliferative DR; OU values represent the mean of right and left eye measurements for each participant; SD: standard deviation; *p* * < 0.05).

**Table 9:** Changes in FAZ Values.

	Group	Stage I–II		Stage III–IV		t	*p*
		Mean (mm^2^)	S.D.	Mean (mm^2^)	S.D.		
FAZ S OU	G1	418.72	132.72	424.10	81.89	−0.119	0.906
	**G2**	**357.37**	**32.25**	**408.11**	**46.71**	**−2.14**	**0.032**
	G3	431.09	92.35	457.61	132.22	−0.491	0.629
	G4	442.66	152.05	467.67	158.75	−0.351	0.73
	G5	426.02	146.96	441.80	163.10	−0.22	0.828
FAZ D OU	G1	431.33	114.55	443.89	76.45	−0.236	0.816
	**G2**	**374.63**	**50.40**	**477.16**	**112.39**	**−2.842**	0.011 *
	G3	437.66	96.29	442.92	128.28	−0.099	0.923
	G4	431.34	100.28	491.02	180.19	−0.948	0.356
	**G5**	**390.12**	**50.90**	**452.34**	**50.49**	**−2.67**	0.027 *

(FAZ S OU: Foveal Avascular Zone-Superficial-Oculus Uterque; FAZ D OU: Foveal Avascular Zone-Deep-Oculus Uterque; mm^2^: square millimeters; G1: systemically healthy group; G2: patients with diabetes mellitus without diabetic retinopathy (DR); G3: patients with non-proliferative DR without diabetic macular edema; G4: patients with non-proliferative DR with diabetic macular edema; G5: patients with proliferative DR; OU values represent the mean of right and left eye measurements for each participant; SD: standard deviation; *p* * < 0.05).

## Data Availability

The data that support the findings of this study are available from the corresponding author upon reasonable request.

## Supplementary Materials

The following supporting information can be downloaded at: https://www.mdpi.com/article/10.3390/diagnostics16050654/s1, Supplementary Table S1: Changes in HbA1c values.

## Abbreviations

DR	Diabetic retinopathy
DM	Diabetes mellitus
OCT	Optical coherence tomography
OCTA	Optical coherence tomography angiography
CSI	Choroid-sclera interface
GCL	Ganglion cell layer
RNFL	Retinal nerve fiber layer
FAZ	Foveal avascular zone
ILM	Inner limiting membrane
PI	Plaque index
GI	Gingival index
PPD	Probing pocket depth
CAL	Clinical attachment loss
RTEU	Recep Tayyip Erdoğan University
AGEs	Advanced glycation end products
TNF	Tumor necrosis factor
IL	Interleukin
