# Supplementary material for: The Effect of Periodontitis Severity on Diabetic Retinopathy: An Optical Coherence Tomography Study
Source: Diagnostics (Basel). 2026 Feb 24;16(5):654. doi: 10.3390/diagnostics16050654 (PMC12984936; doi:10.3390/diagnostics16050654)
Supplement: Supplementary file 1 [file diagnostics-16-00654-s001.zip › diagnostics-4121574-supplementary.pdf]

	Group	Stage I-II		Stage III-IV		t	p
		**Mean (%)**	**S.D.**	**Mean (%)**	**S.D.**		
HbA1c	G1	5	0.3	5.3	0.4	-1.371	0.187
	**G2**	**6.2**	**0.4**	**7.9**	**1.7**	**-3.279**	**0.005****
	G3	9.1	2.1	8.8	2.2	0.337	0.74
	G4	8.5	2.2	8.6	2.1	-0.107	0.916
	G5	9.3	2.5	9.2	1.7	0.089	0.93

(G1: systemically healthy group; G2: patients with diabetes mellitus without diabetic retinopathy (DR); G3: patients with non-proliferative DR without diabetic macular edema; G4: patients with non-proliferative DR with diabetic macular edema; G5: patients with proliferative DR; HbA1c measured by National Glycohemoglobin Standardization Program (NGSP)-certified method; %: percentage; SD: standard deviation; p<0.05).*
